# Editorial: Longitudinal aging research: Cognition, behavior and neuroscience

**DOI:** 10.3389/fnhum.2022.1002560

**Published:** 2022-10-14

**Authors:** Lutz Jäncke, Mike Martin, Christina Röcke, Susan Mérillat

**Affiliations:** ^1^Division of Neuropsychology, Department of Psychology, University of Zurich, Zurich, Switzerland; ^2^University Research Priority Program “Dynamics of Healthy Aging”, University of Zurich, Zurich, Switzerland; ^3^Neuroscience Center Zurich, University of Zurich and ETH Zurich, Zurich, Switzerland; ^4^Center for Gerontology, University of Zurich, Zurich, Switzerland

**Keywords:** aging research, healthy aging, longitudinal aging research, brain imaging, cognitive neuroscience

This Research Topic (RT) covers papers presented at the 5th International Conference on Aging & Cognition in Zurich (https://eucas.org/ac2019/). A particular focus of this RT is placed on longitudinal research, which is still underrepresented compared to the abundantly available cross-sectional studies. Longitudinal studies are, however, urgently needed to understand aging better. A further emphasis is placed on understanding “healthy aging,” thus excluding issues related to dementia, mild cognitive impairments, or clinically relevant limitations in other behavioral domains. Sixteen manuscripts have been accepted for final publication. Seven papers investigated longitudinal changes concerning neuroanatomical, neurophysiological, and psychometric aging issues (Jockwitz et al.; Lacker et al.; Lannen et al.; Sele et al.; Wank et al.; Oschwald et al.; Wehrle et al.). Six papers describe training and intervention effects in older adults regarding cognitive abilities and brain activations (de Haan et al.; Dziemian et al.; Figeys et al.; Gajewski et al.; Kray et al.; Mikos et al.). The remaining three papers report additional age-related findings, either from cross-sectional or methodological studies (O'Connor et al.; Ramanoël et al.; Rieker et al.).

First, we shortly describe the longitudinal contributions included in this RT. The papers by Sele et al. and Oschwald et al. used data from the Longitudinal Healthy Aging Brain (LHAB) database project (Zöllig et al., 2011; Jäncke et al., 2020), an ongoing prospective, longitudinal study conducted at the University of Zurich (Switzerland). Both studies present and discuss the results of the first 4 years of this project. Sele et al. analyzed volumetric cortical and subcortical changes based on T1-weighted MRI scans. Using sophisticated statistical modeling approaches, the authors demonstrated highly correlated change slopes across large parts of the cerebral cortex. At the same time, some brain regions (i.e., medial temporal regions) deviated from this homogeneity and showed an accelerated decline with advancing age. Concerning subcortical regions, change trajectories were highly variable, with some areas showing strong (e.g., hippocampus) and others slight decline (e.g., pallidum).

Oschwald et al. focused on the white matter microstructure and identified age-related fractional anisotropy declines for selected fiber tracts. Although clear declines were also evident for motor ability measures, these changes were only sparsely related to the anatomical changes. Jockwitz et al. report a cross-validation analysis comprising longitudinal data of the 1000BRAINS project conducted at the Research Center Juelich (Germany) and the LHAB project in Zurich. The authors demonstrate that annual percentage changes in cortical thickness revealed similar results for the two independent samples. Between-sample differences were only marginally present after correction for major covariates. This suggests that the development over time is generalizable over independent samples with similar demographic characteristics when the same methodology is used. Lannen et al. and Wehrle et al. describe study designs of two extremely long-lasting (60 years!) retrospective and prospective longitudinal studies conducted in Zurich (Switzerland). The two reports comprise the applied methods, description of cohorts, statistical analysis, ethical problems, data reconstruction, and data validation.

Lacker et al. focus on longitudinal changes over 4 years of steroid hormone concentration (i.e., testosterone, estradiol, or cortisol), genetic markers, psychological wellbeing, and physical health in men between 40 and 75. They identified that psychological wellbeing and physical health remained stable over time. Further, they revealed that estradiol moderated the course of psychological wellbeing, while the androgen receptor gene polymorphism moderated the course of physical health. These results suggest that the hypothalamic-pituitary-gonadal (HPG) axis is essential for maintaining psychological wellbeing in men. At the same time, physical health depends more on interindividual differences in the androgen receptor gene and testosterone. Wank et al. provide short-term longitudinal data based on ambulatory assessment technologies. They studied real-world conversations over 4 days in a sample of cognitively normal older adults using a smartphone application known as the Electronically Activated Recorder (EAR). Specifically, they extracted instances where the participants shared memories and future thoughts, scoring them for their make-up of episodic and semantic detail. They revealed that older age was associated with reduced real-world sharing of autobiographical episodic and semantic memories. In addition, autobiographical episodic memories of older people appeared to be less detailed.

This group of longitudinal studies increases our understanding of how brain anatomy, brain activation, behavior, and endocrinology change in the context of healthy aging. Beyond this, the studies highlight the importance of longitudinal datasets that comprise more than two measurement occasions to enable the advanced analysis of within-person change trajectories. Furthermore, they emphasize the necessity of combining and cross-validating findings across different research groups using different samples (Oschwald et al., 2019). The study design of the ZLS project will provide extraordinary insights into age-dependent behavioral, emotional, and cognitive changes across a very long observation period.

The next group of papers in this RT addresses training and intervention effects in older adult samples regarding cognitive abilities and associated brain activations. Kray et al. applied a pretest-training-posttest design with eight training sessions. Two groups of older adults were either trained in task switching (treatment group) or in performing single tasks (control group). A group of untrained younger adults served as an additional control group. The authors observed training-specific improvements in task switching and training-specific effects at the neuronal level measured with electroencephalography.

Older adults trained in cue updating while switching showed a reduction in mixing costs indicated by the cue-related P3 component and the associated P3 topography. These results are taken as support for an improvement in preparatory updating processes. However, they did not obtain training-specific improvements in the context updating at the behavioral or neuronal levels, suggesting that there are limitations in the transfer of cognitive training. In the training study of Gajewski et al., participants completed a 16-weeks-lasting training intervention during which they practiced various cognitive functions (attention, verbal skills, and executive functions). Compared to active and passive control groups, cognitive training enhanced performance in several cognitive tests dissimilar to the intervention, thus providing some evidence for transfer. A transfer to daily activities was not observed. Dziemian et al. conducted a longitudinal diffusion tensor imaging study to investigate the effects of an intensive, adaptive 4 week-lasting working memory training on white matter microstructure in young and older adults. The authors quantified the anatomical changes using global and tract-wise mean diffusivity measures. As expected, the behavioral analysis showed increased working memory performance after the working memory training. The neuroanatomical analysis revealed decreased mean diffusivity in the older working memory training group after the training intervention in some brain areas (the right inferior longitudinal fasciculus and right superior longitudinal fasciculus). Thus, this study suggests that the behavioral training gains are associated with training-induced changes in white matter integrity in fiber tracts known to underlie working memory performance.

Mikos et al. analyzed whether cognitive training is accompanied by changes in task-induced deactivation within the default mode network (DMN). They analyzed data collected from a group of healthy older adults who completed a 6 week, process-based, object-location memory (OLM) training and an active control group. As a main result, the authors identified that the dorsal DMN was deactivated during the in-scanner task relative to a visual fixation baseline. In addition, this deactivation was more pronounced in the OLM training group relative to the active control group. Many studies have shown that cognitive and motor learning strongly depends on the evaluation of feedback information.

However, the ability to learn from feedback declines with age. Since the neural mechanisms underlying this age-related change in feedback processing are still insufficiently understood, de Haan et al. conducted a study to examine the relation between learning-related neural processes and feedback-based learning in two age groups. Their results show that less successful learning in older adults corresponds to less effective processing of behavior feedback. At the neural level, activation following positive and negative feedback was less distinctive in older adults due to a smaller feedback-evaluation response to positive feedback in this group. These results suggest that diminished learning performance with age can be attributed to a reduced evaluation of positive feedback and reduced knowledge updating related to changes in choice behavior. In another branch of cognitive training and intervention research, the focus is placed on cognitive augmentation and rehabilitation using non-invasive brain stimulation. In their paper, Figeys et al. comprehensively reviewed the existing literature on transcranial direct current stimulation (tDCS) on cognitive performance and hemodynamic response patterns measured with near-infrared spectroscopy (NIRS). They applied rigorous literature research to identify eight studies using simultaneous tDCS and NIRS in older adult samples. Based on these studies, the authors concluded that tDCS effects on cognitive performance and functional NIRS metrics are most prominent in young, healthy adults and appear to become less robust with age. At the same time, the authors are cautious in drawing firm conclusions due to the small number of studies.

In summary, these training and intervention studies align with pertinent literature (Guye et al., 2020) and advance our understanding of the training-induced neural underpinnings using various methods (EEG, resting-state fMRI, diffusion tensor imaging, and fNIRS). At the same time, those studies emphasize the need for interventions that augment far transfer and transfer to activities of daily living (Sala and Gobet, 2019).

The final set of contributions for this RT comprises three papers reporting age-related cross-sectional or methodological studies (O'Connor et al.; Ramanoël et al.; Rieker et al.). Ramanoël et al. examined how older adults process visuospatial information. In their functional magnetic resonance imaging (fMRI) study, young and healthy older subjects performed a landmark-based navigation task while the associated hemodynamic responses were measured. The authors found that the older adults' navigational abilities were overall diminished compared to young adults. Most importantly, the left inferior temporal gyrus and the left amygdala/hippocampus region were less activated during landmark-based navigation in older compared to younger subjects. This between-group difference only appeared when the landmark condition was contrasted with the control condition. They suggest that in older age, fine-grained information processing in occipital and temporal regions is reduced, thus hindering the capacity to use landmarks adequately for navigation. This work supports a better understanding of the neural dynamics subtending landmark-based navigation and provides new insights on the impact of age-related visuospatial processing differences on navigation capabilities.

Rieker et al. focus on the positive impact of bilingualism on cognitive aging. Bilingualism has been regarded as a complex learning experience that might stimulate and support executive functions. Rieker et al. report a study examining bilingual and monolingual older adults performing task-switching under explicit task-cueing vs. memory-based switching conditions. Their results indicate that lifelong bilingualism can promote a flexible adjustment to environmental cues in older adults, but only with increased task demands.

Modern studies on aging must rely on sufficiently large and complex datasets, particularly when collecting neurophysiological data. Consequently, data analysis becomes more and more challenging. O'Connor et al. propose a sophisticated functional principal component analysis (PCA) method for analyzing large-scale datasets. They applied this technique to NIRS data, which measured cerebral oxygenation during standing in a large study cohort (The Irish Longitudinal Study on Aging, TILDA). The application of fPCA elegantly reduced a large amount of data to a few parameters, summarizing the inter-participant variation of the obtained data during standing. Thus, this technique has been proven useful for data-driven approaches to high-resolution data analysis in aging research.

## Conclusion

This RT provides a comprehensive overview of the current approaches to studying cognitive, neurophysiological, and neuroanatomical aging. The longitudinal studies show that aging is accompanied by specific neuroanatomic and neurophysiological changes. These studies have also emphasized the importance of collecting longitudinal data across more than two time points. In addition, evidence is provided that the identified changes are robust and consistent across different study samples. The Zurich Longitudinal Studies (ZLS) study protocols, which comprise an outstanding long observation period, promise essential insights into the lifelong cognitive aging processes. The intervention and cross-sectional studies emphasize that the aging brain and the associated behaviors can be influenced by training. Therefore, we hope this RT will prompt a more positive view of the opportunities and resources linked with healthy aging. In addition, we hope that this research area will attract more researchers from related disciplines such as biology, psychology, neurology, psychiatry, and public health. Strengthening interdisciplinary collaboration will improve our understanding of the complex human aging process and facilitate innovation to support a healthy aging population (Cherbuin et al., 2021).

## Author contributions

LJ, MM, CR, and SM writing of the manuscript. All authors contributed to the article and approved the submitted version.

## Conflict of interest

The authors declare that the research was conducted in the absence of any commercial or financial relationships that could be construed as a potential conflict of interest.

## Publisher's note

All claims expressed in this article are solely those of the authors and do not necessarily represent those of their affiliated organizations, or those of the publisher, the editors and the reviewers. Any product that may be evaluated in this article, or claim that may be made by its manufacturer, is not guaranteed or endorsed by the publisher.
